# EMS mutation and SNP detection in intracellular *Wolbachia* genomes

**DOI:** 10.1128/msystems.00660-26

**Published:** 2026-07-06

**Authors:** Gabriel Penunuri, Evan Pepper-Tunick, Jakob McBroome, Russell Corbett-Detig, Shelbi L. Russell

**Affiliations:** 1 Department of Biomolecular Engineering, University of California Santa Cruz 8787, Santa Cruz, California, USA; 2 Genomics Institute, University of California Santa Cruz 8787, Santa Cruz, California, USA; 3 Institute for Systems Biology 7268, Seattle, Washington, USA; 4 Molecular Engineering and Sciences Institute, University of Washington 7284, Seattle, Washington, USA; University of Minnesota Twin Cities, Minneapolis, Minnesota, USA

**Keywords:** *Wolbachia*, EMS, intracellular genomes, mutation spectra, high-fidelity sequencing

## Abstract

Endosymbiotic bacteria such as *Wolbachia* pose significant challenges to genetic and molecular investigation due to their obligate intracellular lifestyle and complex growth requirements. Current understanding of their protein biology relies heavily on functional assignments inferred by homology, which may not reflect the specific roles endosymbiont proteins play within the host. This work addresses the need for robust genetic perturbation by demonstrating the successful application and detection of chemical mutagenesis in the genome of the *w*Mel strain of *Wolbachia* grown within a stably infected *Drosophila melanogaster* JW18 cell line. To accurately detect ethyl methanesulfonate (EMS)-induced mutations in a large, unsorted cell culture population, in which mutations remain at very low allele frequency, we implemented an ultra-low error rate sequencing strategy, circle sequencing. This technique enables confident detection of EMS-induced single-nucleotide polymorphisms (SNPs) that would be swamped by the inherent error rates of standard next-generation sequencing. Circle sequencing library preparations successfully revealed a clear EMS mutation signal in treated cells, characterized by a significant enrichment of canonical C/G > T/A transitions. Furthermore, we present a model explaining observed EMS mutation rates across the genome for different sequence contexts. These findings show that EMS-treatment can successfully leave detectable mutation signals in intracellular genomes and offer promise for the future development of protocols to make targeted edits in *Wolbachia* genomes.

**IMPORTANCE:**

As the use of intracellular symbionts for bioengineering projects grows, so does the need for foundational protocols for the genetic manipulation of intracellular genomes. Ethyl methanesulfonate (EMS), a chemical mutagen, has been a research tool for initial genomic analysis of gene function in plant and animal systems for decades and represents an established way of generating mutations for future functional testing. This study demonstrates that EMS can be used to induce mutations in the genome of the unculturable symbiont Wolbachia. This approach may help overcome a key barrier in Wolbachia biology—namely, the lack of forward genetic tools—and could provide a foundation for genetic manipulation of intracellular symbionts, including those used in pest and disease control.

## INTRODUCTION

Interest in harnessing intracellular symbiont for large-scale biological control continues to grow, particularly as *Wolbachia* demonstrates effectiveness in controlling mosquito-borne diseases and arthropod pest populations (1, 2). Because of active bioengineering development around obligate intracellular symbionts, particularly *Wolbachia*, accurate functional annotation of endosymbiont genes is critical for progress. Current functional assignments are frequently inferred through homology to genes in free-living organisms, which may not accurately reflect their specific roles within the symbiotic context (3).

The absence of robust methodologies for *Wolbachia* genetic perturbation severely constrains the identification of essential genes and the elucidation of specific gene functions (4). Intracellular bacteria present significant obstacles to comprehensive investigation; their obligate lifestyle requires complex growth conditions that make genome-wide approaches difficult to scale (5, 6). Even within stable growth conditions, researchers have found intracellular microbes extremely difficult to genetically manipulate, greatly limiting the scope of experimental interventions (4). While impressive advances have been made in obligate intracellular pathogens, such as *Rickettsia* (7) and *Chlamydia trachomatis* (8–10)*,* progress is slow and expensive. With interest in obligate intracellular symbionts such as *Wolbachia* continuing to increase, the development of a high-throughput, genome-wide random mutagenesis methodology would be useful to establishing foundational functional genetic research.

Chemical mutagenesis with ethyl methanesulfonate (EMS) has been a foundational tool in genetics for over a century, enabling systematic functional genetic discoveries across a wide range of plant and animal systems (11). In contrast, the application of EMS to obligate intracellular bacteria has been limited, with only a small number of studies reporting genome-wide detection of EMS-induced single-nucleotide polymorphisms (SNPs) (4, 9, 10, 12). A primary barrier has been that EMS induces mutations at frequencies three to four orders of magnitude lower than the intrinsic error rates of standard next-generation sequencing platforms (13–16). This disparity between mutation frequency and technical detectability has prevented the use of EMS in intracellular microbes despite its proven value for functional genetic analysis in nearly all other biological contexts. Overcoming this limitation requires sequencing approaches capable of substantially suppressing technical error in order to reveal rare, chemically induced mutations.

Here, we overcame these technical barriers by combining chemical mutagenesis with high-fidelity sequencing in a tractable intracellular bacterial system. We leverage a *Drosophila melanogaster* cell line stably infected with the *Wolbachia w*Mel strain, which permits controlled chemical perturbation of the endosymbiont genome. To enable reliable detection of rare EMS-induced variants, we implement circle sequencing (17, 18), a low-error rate library preparation strategy that generates consensus sequences from repeated reads of individual DNA molecules, thereby reducing sequencing artifacts well below standard Illumina error rates. Using this approach, we demonstrate clear detection of EMS mutagenesis signals in treated cell cultures, including the enrichment of canonical EMS-associated substitutions and non-random sequence context biases in mutation positions. By demonstrating the first intentional mutagenesis and high-confidence variant detection in *Wolbachia*, this approach establishes a critical prerequisite for future targeted genome editing in these endosymbionts.

## MATERIALS AND METHODS

### Cell lines

We maintained *Wolbachia w*Mel-infected immortalized JW18 (19) *Drosophila melanogaster* cell lines in 4 mL of media composed of Sheilds and Sang M3 medium (Sigma S3652) and 10% (vol/vol) fetal bovine serum (Gibco A3160502) in plug-seal T25 flasks (Corning 430639), incubated at 25–26°C (as described in reference 20).

Cell cultures were maintained on a 7-day schedule, splitting at a 1:2 ratio for *w*Mel-infected lines and 1:6 to 1:4 ratio for uninfected lines. For cell passaging, we removed old media, added fresh media, and then dislodged adherent cells by scraping the flask surface with a sterile bent Pasteur pipette (Fisher, 1367820D). Resuspended cells were transferred at the appropriate ratio to new T25 flasks, and the volume was adjusted to 4 mL with fresh media. Infection states were monitored with fluorescent *in situ* hybridization against *w*Mel 16S ribosomal RNA and whole-genome sequencing (as in reference 21).

### EMS treatment

We first quantified the *w*Mel-infected JW18 cell concentration in culture with a hemocytometer and mixed media with cells with fresh media in T25 flasks for a final concentration of 1.69e6 cells/mL. These cells were incubated at 26°C for 1 day prior to treatment.

To create EMS-treated and control samples, we added either 20 µL of fresh 0.5% (vol/vol) EMS (Sigma M0880) or mock-treatment media, respectively, to 3,980 µL of Shields and Sang M3 media supplemented with 10% FBS and sterilized through a 0.2 µm syringe filter. We then removed and replaced the old media from previously quantified cell cultures with either EMS or mock media. Flasks were then incubated at 25–26°C for 3, 5, or 7 days.

### Library preparation and sequencing

We obtained cell pellets from the EMS-treated and controlled cell cultures by scraping the flasks, pipetting 1 mL into 1.5 mL epi tubes, and pelleting the cells by centrifugation at 10,000 × *g* for 3 min at 4℃.

We amplified genomic DNA templates by rolling circle amplification (RCA) using the procedure described in the initial circle sequencing publication by Lou et al. (17), with several adjustments. DNA purification steps were done with a SPRI magnetic bead protocol instead of the QIAGEN MinElute clean up kit. We sheared gDNA resuspended in TE buffer over two rounds of 5 min with a spin down between rounds. We set the duty cycle to 30%, peak power to 60 W, and 1,000 cycles per burst. Phosphorylation was done with the T4 PNK as described in the supplemental methods of Lou et al. (17). The DNA was then denatured and flash frozen according to the Lou et al. protocol. For the circularization reactions, we used the same master mix except we added no more than 3.5 pmol of ssDNA instead of 5 pmol. Linear DNA remaining in the reaction mix was digested according to the Lou et al. protocol. For the rolling circle amplification (RCA) reactions, we skipped the random primer annealing reaction and instead used 2.5 μL of 10 μM random hexamer primers in the RCA reaction mix. We used 2.5 μL (instead of 1 μL) of 10 mM dNTP and did not include the inorganic pyrophosphatase. After performing RCA, we treated about 20–30 ng of RCA DNA products with 1 μL of DNA Exonuclease I (NEB M0293S) and 0.5 μL of DNA Exonuclease III (NEB M0206S) for 1 h at 37°C to digest linear fragments. We then purified samples using SPRI magnetic beads and Qubit quantified with the dsDNA HS Assay Kit (Thermo Fisher Q32851).

We prepared libraries from the RCA products by mixing 10 ng of RCA products with 2 μL charged Tn5 transposase (Tn5 enzyme was expressed and purified in-house) in 12 μL of molecular grade water and 4 μL of 5× TAPS-PEG 8000 for 8 min at 55°C (22). The reaction was halted by adding 0.2% SDS and incubated at room temperature for 7 min. We set up PCRs using the KAPA Biosystems HiFi Polymerase Kit (Roche) and amplified for 12 cycles using uniquely indexed i5 and i7 primers. We ran these PCR products on a Thermo Fisher 2% E-Gel EX Agarose Gel (G401002) and cut between ~400 and 700 bp. The gel was purified with the NEB Monarch DNA Gel Extraction Kit (NEB #T1020) and quantified using the dsDNA HS Assay Qubit Kit and the Agilent TapeStation D1000 tapes (5,067–5,582). We prepared two pools of circle sequencing Tn5 libraries: one pool containing 14 purified and indexed libraries from 5-day samples, and a second pool containing 14 purified and indexed libraries from 3- and 7-day samples. We sequenced both pools using 150-bp paired-end reads across two Illumina HiSeq 4000 lanes at Fulgent Genetics.

### Circle sequencing processing

We processed the demultiplexed circle sequencing short read data using a custom snakemake (23) pipeline (https://github.com/jmcbroome/circleseq). We aligned paired-end sequencing FASTQ reads to the *w*Mel *Wolbachia* reference genome (NC_002978.6) using BWA-MEM (24) and output the alignments as SAM files. The aligned sequences were divided into repeat subsequences based on their mapping, and trimmed repeat sequences separated from the primary alignment with custom python scripts (25). We selected and extracted reference genome regions with BEDTools getfasta (25, 26) based on the genomic coordinates of the primary alignments generated from the BWA mapping step. Groups of subsequences were then realigned to their mapping region using an optimized striped Smith-Waterman alignment provided by scikit-bio (25). For each group, we constructed a consensus sequence from the realigned subsequences and defined the quality score as the number of times the base was observed. Any base with a mismatch in a subsequence realignment was replaced with an N. In the final step, the consensus alignments were piled up with SAMtools mpileup (27), after filtering positions with less than two subreads present.

This approach, although sharing the same core idea of implementing error-suppression with consensus construction, differs slightly from the original methods published by Lou et al. (17). We have opted to use reference-anchored repeat detection through BWA-MEM alignment instead of self-alignment between paired-end reads. Our approach also differs by how the consensus is built. Originally published methods did so from explicit tandem copies where each repeat corresponds to a single pass around the circularized read leading to repeat boundaries that are regular and periodic. Our approach builds the consensus from subsequences extracted after mapping, resulting in length variation that is more tolerant of irregular repeat structure which can better reflect artifacts of Illumina library preparation. We also implement a two pass alignment strategy that allows for greater specificity and scalability for genome-wide analysis through performing a local Smith-Waterman realignment after the initial global BWA-MEM alignment. This step further builds analysis tolerance for truncated repeats, uneven coverage, and sequencing dropouts that would all be expected from Illumina short-read sequencing. It is also during this secondary alignment step that we increase the specificity of variant detection and lower the false-positive rate. We are able to track any disagreement across the subreads and mark these potential PCR-induced errors or alignment artifacts by calling the bases as N removing them from downstream analysis.

### Mutation rate estimation

#### Per-sample rates

We collected mutation counts from mpileup files with a custom python script (https://github.com/gabepen/ems_effect). We loaded site-level mutation counts from these mpileup files. Sites were restricted to G/C positions. For C sites, only C→T mutations were counted; for G sites, only G→A mutations. We constructed an exclusion mask representing genomic positions where mutated sites appeared in more than one control sample. We used this mask to exclude these positions from all downstream analysis. We extracted sequence contexts (3mers, 5mers, and 7mers) from the reference genome, and G-centered contexts were reverse-complemented to C-centered for canonicalization. We used a finite-sites model to estimate an initial per-sample mutation rate for mutation spectra visualization:


(1) $${\hat{\mu }}_{\text{high}}=\frac{A_{\text{total}}}{D_{\text{total}}}$$

where *A*_total_ is the count of alternative alleles across all positions and *D*_total_ is the total sequencing depth across all sites.

We modeled per-sample mutation rates using generalized linear models (GLMs) with log(depth) as an offset starting with a background subtraction:


(2) $$Y_{i,\text{adj}}=\max(0,Y_{i,\text{raw}}-\alpha \cdot d_{i})$$

These adjusted counts are then used in a GLM:


(3) $$\log(E[\max(0,Y_{i,\text{raw}}-\alpha \cdot d_{i})])=\log(d_{i})+\beta_{0}$$

where *Y_i_*_,raw_ is the raw mutation count at a site *i, Y_i_*_,adj_ is the alpha-adjusted mutation count at a site *i, d_i_* is the sequencing depth at site *i, α* is the background false positive rate (estimated from controls), and *β*_0_ is the intercept parameter, estimated per sample.

We then estimated rates as follows:


(4) $${\hat{\mu }}_{s}=\exp({\hat{\beta }}_{0,s})$$

### Mutation rate estimation across genomic categories

We estimated mutation rates for intergenic, synonymous, and non-synonomous rates with a negative binomial GLM. We opted to use a negative binomial distribution to account for the overdispersion and low mutation counts in the finite-sites data set. We fit this model with treatment and category covariates, including interactions:


(5) $$\begin{array}{rl} \log(E[Y_{i}]) & =\beta_{0}+\beta_{\text{treatment}}\cdot T_{i}+\beta_{\text{syn}}\cdot S_{i}+\beta_{\text{nonsyn}}\cdot N_{i}\\ & \;+\beta_{\text{treatment}\times \text{syn}}\cdot T_{i}\cdot S_{i}+\beta_{\text{treatment}\times \text{nonsyn}}\cdot T_{i}\cdot N_{i}+\log(d_{i}) \end{array}$$

We modeled these same genomic categories with an alternative approach to capture the absolute instead of the proportional effect of treatment on category rates starting with a control only model to establish a baseline:


(6) $$\log(E[Y_{i}^{\text{control}}])=\beta_{0}^{\text{control}}+\beta_{\text{syn}}^{\text{control}}\cdot S_{i}+\beta_{\text{nonsyn}}^{\text{control}}\cdot N_{i}+\log(d_{i})$$

Next, we calculated the expected mutation excess with treatment:


(7) $$Y_{\text{excess}}[i]=Y_{\text{observed}}[i]-E[Y_{i}^{\text{control}}]$$

We then fit two GLMs using Gaussian family with an identity link, the first a model for constant absolute effect:


(8) $$E[Y_{\text{excess}}[i]]=\beta_{\text{treatment}}\times d_{i}\times T_{i}$$

And second an interaction model:


(9) $$\begin{array}{rl} E[Y_{\text{excess}}[i]] & =\beta_{\text{treatment}}\cdot d_{i}\cdot T_{i}\\ & \;+\beta_{\text{treatment}\times \text{syn}}\cdot d_{i}\cdot T_{i}\cdot S_{i}\\ & \;+\beta_{\text{treatment}\times \text{nonsyn}}\cdot d_{i}\cdot T_{i}\cdot N_{i} \end{array}$$

We also estimated category rates for treatment time with a negative binomial GLM, where no-label is the 5-day reference:


(10) $$\log(E[Y_{i}])=\beta_{0}+\beta_{3d}\cdot D_{3d,i}+\beta_{7d}\cdot D_{7d,i}+\log(d_{i})$$

### Mutation sequence context analysis

We modeled per-site EMS mutation counts using generalized linear models (GLMs) with sequence-context features.

Site-level mutation counts were loaded from mpileup files. Sites were restricted to G/C positions. For C sites, only C→T mutations were counted; for G sites, only G→A mutations. Sites in an exclusion mask (those appearing in multiple control samples) were excluded. Sequence contexts (3mers, 5mers, and 7mers) were extracted from the reference genome, and G-centered contexts were reverse-complemented to C-centered for canonicalization.

We used Negative Binomial GLMs to model the sequence context of EMS mutations. The per-site EMS mutation count served as the response variable, and log(depth) was included as an offset term. The model evaluated the effect of the experimental conditions by including both the treated and control groups as primary covariates.


(11) $$\log(E[Y_{i}])=\beta_{0}+\beta_{\text{treatment}}\cdot T_{i}+\sum_{j}\beta_{j}\cdot X_{i,j}+\log(d_{i})$$

where *Y_i_* is the raw mutation count at a site *i, T_i_* is the treatment indicator, *X_i,j_* represents sequence context features (vary by model type), *d_i_* is the sequencing depth at site *i, β*_treatment_ is the treatment effect on log scale, *β*_*j*_ represents sequence context coefficients, and *β*_0_ is the intercept parameter, baseline log rate for control sites.

To account for sequence context dependencies and to determine what context was the best predictor of mutation bias, we implemented five distinct encoding schemes.

1. Positional: We encoded sequence context using 12 binary features representing the base identity (T, G, or C) at each of the 4 varying positions in the canonicalized 5-mer, with A as the reference category for each position. The center position is always C, so 3 features are constant; 12 features vary across sites. Coefficients represent log-enrichment relative to A at each position.
2. 3-mer: We encoded context using 16 binary features representing all possible C-centered 3-mers spanning positions −1, 0, 1.
3. 5-mer: We encoded context using a one-hot encoding approach, resulting in 256 binary features. 5-mer refers to the −2, −1, 0, 1, 2 positions with respect to an EMS mutation at position 0.
4. Positional-3-mer: We encoded context using 32 binary features, representing the left and right 3-mers covering the −2, −1, 0 and 0, 1, 2 positions, respectively. This model also evaluates C-centered mutations only resulting in fixed “0” positions in both left and right 3-mers.
5. 7-mer: We encoded context using a one-hot encoding approach, with a theoretical maximum of 4,096. 7-mer refers to the −3, −2, −1, 0, 1, 2, 3 positions with respect to an EMS mutation at position 0. To manage memory usage, the model required the input contexts to be split into separate groups deterministically by first sorting unique 7-mers. Model fitting was performed independently for each split, and the resultant metrics were subsequently aggregated to obtain the final summary statistics.

After fitting, we predicted mutation counts for each site under control and treated conditions. We converted predicted counts to per-base mutation rates by dividing by depth and then aggregated observed and predicted rates by the relevant sequence-context category for the model using depth-weighted means. We evaluated fit by comparing observed vs predicted aggregated rates using scatter plots and correlation metrics.

### Cross-validation evaluation (out-of-sample)

We used fivefold cross-validation to measure out-of-sample predictive performance. We split sites into fivefold with sklearn.model_selection.KFold using shuffling (random_state = 42). For each fold, we trained each model on fourfold and evaluated it on the held-out fold, so every site served as test data exactly once. For each held-out fold, we computed mean squared error (MSE), mean absolute error (MAE), Pearson correlation (*r*), and a negative-binomial deviance statistic. We summarized performance across folds by reporting the mean (and, when available, the standard deviation) of each metric.

For the 7-mer split model, we controlled memory usage by fitting the model in groups of 7-mer categories. We deterministically partitioned unique 7-mers into groups using a round-robin assignment over sorted 7-mer categories. Within each cross-validation fold, we fit one GLM per group using only training sites in that group. We generated held-out predictions by applying the corresponding group model to held-out sites in that group (each site received a prediction from exactly one split model), and we computed cross-validation metrics from the concatenated held-out predictions across groups.

### Prediction accuracy metrics (evaluation data set; in-sample in the standard pipeline)

We also evaluated prediction accuracy on the evaluation data set by comparing observed mutation counts (y) to model-predicted expected counts (mu) while incorporating the log(depth) offset. For the 7-mer split model, we produced predictions by routing each site to the split model corresponding to its 7-mer category, and we evaluated performance only on sites with a valid 7-mer context.

We computed MSE, MAE, RMSE, and MAPE; Pearson correlation (*r*) and Spearman correlation (rho) with *P*-values; R-squared and adjusted R-squared; a deviance statistic; MSLE and RMSLE; and median-threshold classification metrics (accuracy, precision, recall, and F1). We also reported the number of evaluated sites and the mean observed and predicted counts.

### Genome-wide EMS mutation enrichment

We analyzed local genomic variation independent of genomic categories by dividing the genome into fixed-size, non-overallaping 5-mer windows and testing if the observed rates deviated from the 5-mer model for EMS mutation context and rate.

For each mutated site *i* in window *W_k_,* we extract the 5-mer context and predict the expected mutation rate using the pre-fitted 5-mer model. We then fit a site-level GLM with a 5-mer offset and a treatment covariate:


(12) $$\log(E[Y_{i}])=\log(\mu_{\text{5mer},i,s})+\log(d_{i})+\beta_{0,k}+\beta_{\text{treatment},k}\cdot T_{i}$$

where *Y_i_* is the observed mutation count at a site *i, T_i_* is the treatment indicator, *μ*_5mer,*i*,*s*_ is the pre-computed expected rate from the 5-mer model, *d_i_* is the sequencing depth at site *i, β*_treatment,*k*_ is the window-specific treatment effect, and *β*_0,*k*_ is the window-specific intercept*.*

### Genome-wide EMS mutation enrichment in the context of transcription

We tested the correlation between EMS mutation enrichment and rates of transcription by adapting the fixed-size window models above to encompass genic regions and performed a regression analysis between the predicted window mutation rates and TPM values for each corresponding gene. To obtain TPM values for the wMel genome, we downloaded the wMel-infected JW18 cell RNAseq data set produced by Jacobs et al. (28) (PRJNA1240446) and regenerated pseudoalignments according to their methods (28). We used ordinary least squares (OLS) linear regression fitting controls and treated samples separately.

## RESULTS

### EMS mutation signal

**Fig 1:**
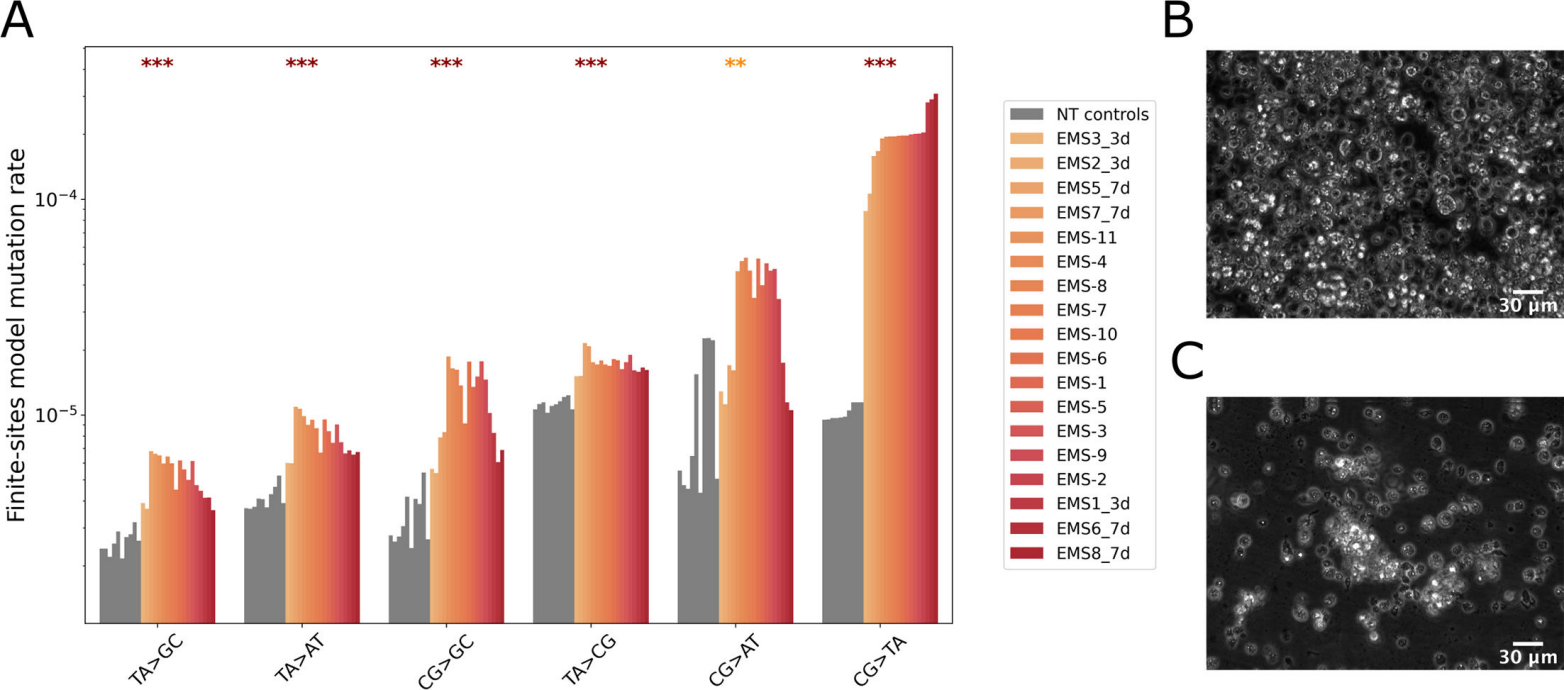
Treating *w*Mel-infected JW18 cells with EMS induces C > T and G > A mutations in the *w*Mel genome. (**A**) EMS mutation spectra demonstrating a large difference between control (gray) and treatment (orange-red gradient by experiment) at the canonical EMS transition types of C > T and G > A. Asterisks denote one-sided Mann–Whitney tests (EMS > NT) on per-sample strand-collapsed finite-sites mutation rates, with Benjamini–Hochberg FDR across the six mutation classes: **, *q* < 0.01; ***, *q* < 1 × 10⁻⁴. Representative tissue culture micrographs of *w*Mel-infected JW18 cells (**B**) after the control treatment and (**C**) after EMS treatment.

Using high-fidelity circle sequencing (17), we identified an elevated mutation rate in the EMS-treated *w*Mel genome cultured in *Drosophila* JW18 cells, relative to control treatments. As Wolbachia exist at high copy numbers within host cells, each sample represents a population of bacteria; accordingly, all mutation analyses are conducted at the population level across the treatment group rather than in individual bacterial genomes. Following Illumina sequencing and demultiplexing of the 18 EMS-treated and 10 control samples, we obtained 4,691,282,072 total reads estimated to be derived from 619,355,451 rolling circle amplification (RCA) products amounting to 2,755,257,101 total read copies (Tables S1 and S2). When we compared EMS-treated and control consensus circle sequencing reads to the *Wolbachia w*Mel reference genome, GCF_000008025.1, we identified four SNPs fixed across all samples and excluded these from the analysis. Treated samples showed a significant enrichment of the canonical EMS-induced transitions C > T and G > A (*P* < 0.001, Mann-Whitney *U* test, Fig. 1A; Fig. S1). These mutations affected the entire genome (Fig. S2; Table S3). We also noted changes to the physical characteristics of the EMS-treated cell lines when compared to the control lines (Fig. 1B and C). Treated cells exhibited morphological signs of stress including slowed growth, small cell size, condensed cytoplasm, and disrupted membranes.

We represented the mutation spectra as a depth-normalized proportion of sites mutated for each transition type using the finite-sites model, indicating that the EMS mutation signal was only clearly detectable through low error rate sequencing methods (Fig. 1A). In previously published mutation rates, EMS has been shown to induce per-site mutation rates of ∼1 × 10^–6^ to ∼1 × 10^–2^ across different organisms (13, 14), while the minimum medium error rate on an Illumina platform is around 5 × 10^–3^ per-site (29). We calculated the error rate of our circle sequencing libraries for different counts of RCA tandem repeats used to correct errors observed on the first repeat. The initial repeat consensus vs reference mismatch rate was 6.297 × 10^–4^ which was reduced to 6.451 × 10^–5^ with the incorporation of a second tandem repeat in the consensus and further down to 3.049 × 10^–5^ with the incorporation of four repeats (Fig. S3).

### EMS mutation rates

**Fig 2:**
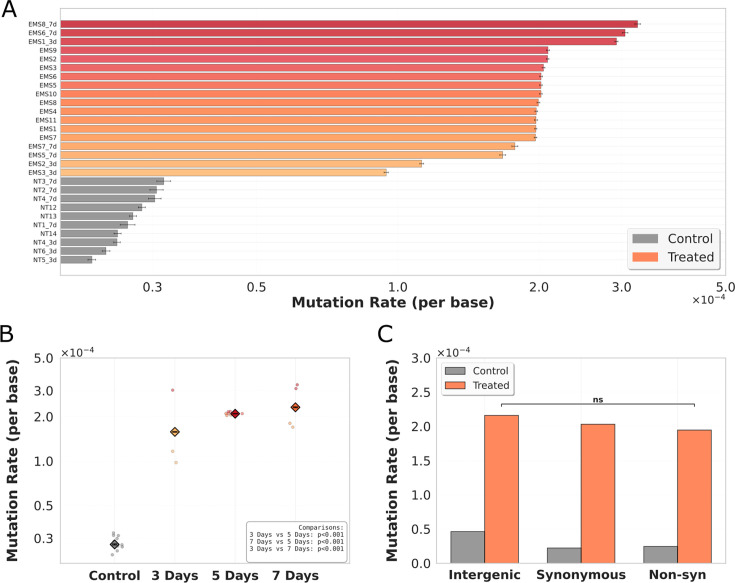
EMS treatment duration was positively associated with mutation rate, whereas functional aspects of the wMel genome exhibited no association. (**A**) Per-sample EMS mutation rates for treated and control samples as estimated by fitting a negative binomial GLM. Treated samples exhibit ~5-fold higher mutation rates than control samples. (**B**) Treatment time had a significant impact on mutation rate when treatment time was modeled as a covariate of mutation rate, with longer treatment times correlating with higher mutation rates. (**C**) Intergenic vs coding mutation rate analysis with treated rates normalized by 5-mer content in each region category. Similar absolute rate differences among coding categories suggest that EMS treatment added the same number of mutations per base pair across genomic categories.

We estimated mutation rates across all samples using site-level GLMs, obtaining estimates of per-sample rates ranging from 2.23 × 10^–5^ to 3.17 × 10^–5^ mutations per base pair for controls (mean: 2.71 × 10^–5^ mutations/bp) and 9.44 × 10^–5^ to 3.24 × 10^–4^ for treated samples (mean: 2.05 × 10^–4^ mutations/bp) (Fig. 2A). Treated samples exhibited significantly elevated mutation rates with average increase of 4.73-fold (95% CI: 4.65–4.80) compared to controls (*P* < 0.001, negative binomial GLM with treatment covariate).

The treatment durations used in the EMS dosage protocol had a proportionate effect on mutation rate when we modeled the interaction between treatment duration and mutation rate using a negative binomial GLM with days of treatment as a covariate. We grouped control and treated samples as 5-day (treated = 11, control = 3), 3-day (treated = 3, control = 3), and 7-day (treated = 4, control = 4). All pairwise comparisons between groups were significant with the group-level estimates showing an increase in mutation rates as treatment time increased (Fig. 2B). However, we note substantial inter-sample variance over each treatment duration category.

We determined that the functional sequence category has a negligible differential effect on EMS mutation incidence by comparing mutation rates across three different functional coding categories: intergenic, synonymous, and non-synonomous. We calculated mutation rates by incorporating a baseline mutation rate for each genomic category using the 5-mer model described in the mutation sequence context analysis portion of the methods. This accounted for any significant divergence in sequence content between coding and intergenic regions. We estimated control mutation rates for these three categories to be 4.63 × 10^–5^, 2.24 × 10^–5^, and 2.26 × 10^–5^ mutations per base, respectively. We estimated treated rates to be 2.16 × 10^–4^ mutations per base pair for intergenic sites, 2.03 × 10^–4^ mutations per base pair for synonymous sites, and 1.95 × 10^–4^ mutations per base for non-synonomous sites (Fig. 2C). Although these rates represent large differences between categories in the proportional changes between the control and treated samples, they amount to similar absolute effect, as quantified by the number of mutated bases added by EMS to the background control rates. For the same three categories, the absolute EMS effect, or mutations per base added by treatment, were 1.16 × 10^–4^, 1.73 × 10^–4^, and 1.62 × 10^–4^. While the constant effect and interaction models produce statistically significant effect differences (*P* < 10^–154^, likelihood ratio test), they only explain about ~5% of the variation. This indicates that the rate differences between intergenic and genic regions are biologically insignificant.

We found limited evidence to suggest that EMS preferentially mutagenizes particular windows of the *w*Mel genome or is more likely to impact regions that are open for transcription. We calculated EMS mutation rates within 2,500 base pair non-overlapping windows across the genome using our k-mer aware genomic window rate model (equation 12) to identify biologically meaningful hot or cold spots. We used a summed EMS mutation count normalized by sequencing depth across all samples as input into the model. We established the genome-wide mean as the average of all window mutation rates calculated by the genomic window rate model (equation 12). A binomial test of window clusters, comparing mutation rates to the genome-wide mean, identified no stretches of EMS hot or cold spots beyond a single window. One such example includes a weak difference between mutation rate of the prophage regions, WO-A and WO-B, and the rest of the genome. The rate ratio between the prophage regions and non-prophage was 0.966 (95% CI 0.961–0.971). Paired per-sample comparisons indicated a consistent prophage-associated reduction in treated samples (median per-sample rate ratio 0.962; Wilcoxon *P* = 5.3 × 10^–5^) but not in the controls (*P* = 0.16). We also calculated EMS mutation rates for each gene and performed a regression analysis with *w*Mel *Wolbachia* gene expression data collected by Jacobs et al. (28). The expression data were very weakly correlated with EMS mutation rate (*R*^2^ = 0.025, Fig. S4). This combined with the random spatial distribution of significantly elevated windows that have apparent clustering suggests that mutation rate variation in this system is influenced by unmodeled effects. While the treatment induces a global increase in mutation rates, the local variation appears to be largely random, with no evidence for transcription-associated mutagenesis.

### EMS mutation sequence context bias

We selected the 5mer model for determining sequence bias in EMS mutations because it provided the best balance between model fit and generalizability across multiple performance metrics (Table S4). The 7mer model achieved the lowest AIC among all evaluated models with a ΔAIC to the next lowest, the 5mer model, of ~18,000 (Table S4). However, the 7mer model also reported the highest overall BIC indicating the capturing of a significant amount of noise. The 5mer model reported the lowest BIC with a ΔBIC of ~5,000 to the second lowest, the positional-3mer. Thus, our prediction accuracy and cross-validation tests supported the 5mer model over the 7mer, 3mer, and positional models (Fig. S5). For prediction accuracy, the 5mer model had the lowest RMSE (0.546) and highest Pearson *R* (0.348) with the positional-3mer model performing second best (RMSE = 0.299, Pearson *R* = 0.343) and the 7mer third (RMSE = 0.300, Pearson *R* = 0.342); while simpler models (positional, 3mer) showed reduced predictive accuracy. Cross-validation (CV) metrics further suggested although the 7mer model was adept at producing rankings, overfitting likely hindered its calibration. The 7mer model had the highest CV-MSE (*R* = 0.319) and lowest CV-Pearson (*R* = 0.339). The 5mer model performed the best in these metrics with *R* = 0.298 and 0.367, respectively.

**Fig 3:**
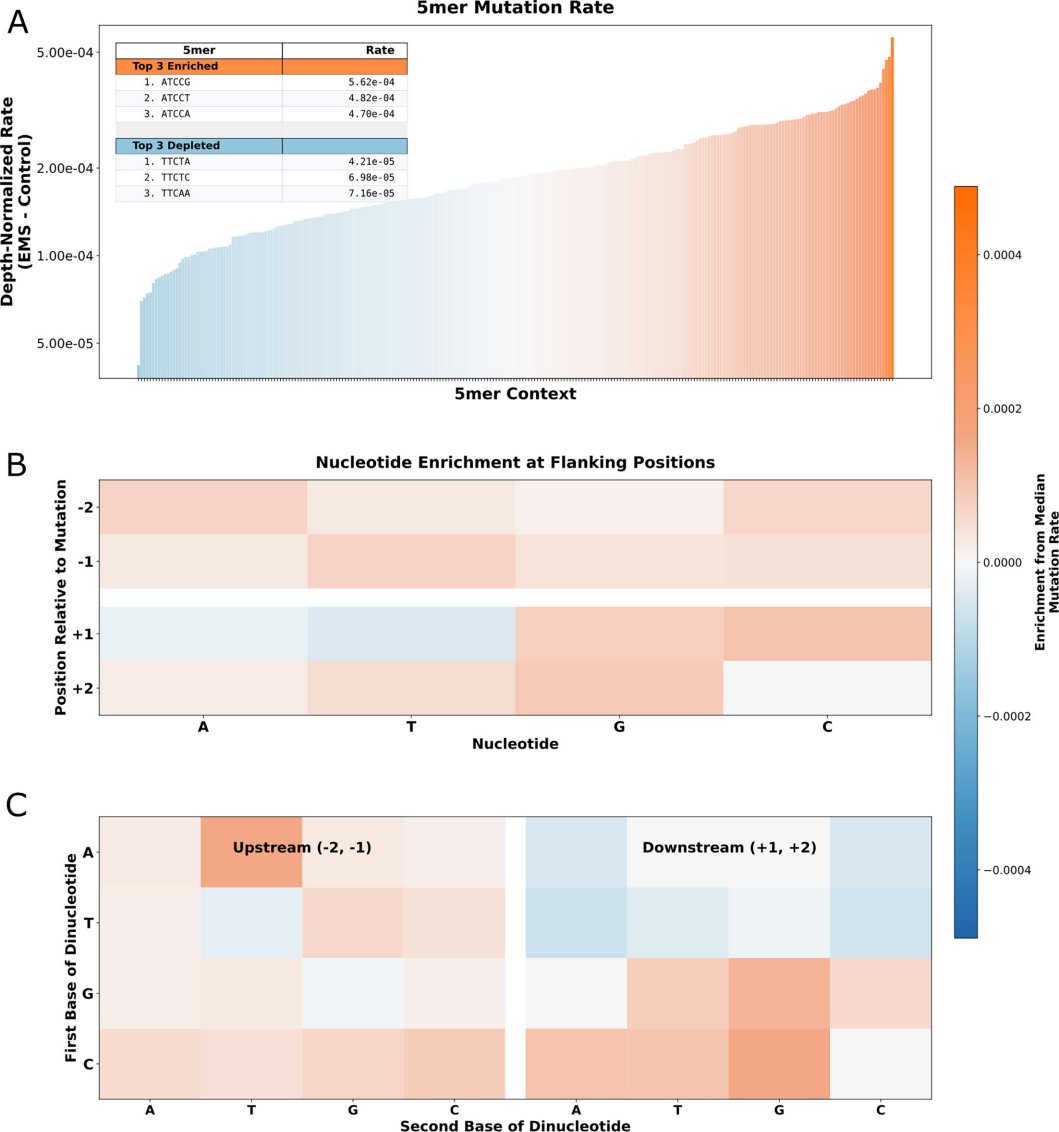
EMS mutations in the wMel genome exhibit biased sequence context. (**A**) Mutation rates of all possible 5mer sequences. Strands are collapsed, such that all canonical EMS mutations are treated as C-centered. The top three enriched and depleted sequences are shown in a table with the associated mutation rate. (**B**) Single-nucleotide enrichment at each of the four positions flanking a canonical EMS mutation site. (**C**) Dinucleotide pair enrichment at the upstream and downstream positions flanking an EMS mutation site.

All models performed poorly at predicting EMS mutations from sequence context alone, indicating that additional factors contribute to EMS mutation bias. Nevertheless, our fitted 5mer model identified significant enrichment of certain sequence contexts at EMS-mutated sites (Fig. 3; Table S5). AT dinucleotides were enriched at the −2 and −1 positions directly upstream of EMS-mutated bases (Fig. 3C), while dinucleotides containing A or T were depleted at the downstream +1 and +2 positions where C or G dinucleotides were enriched. Our results suggest that although C and G bases are enriched near EMS-induced SNPs, this enrichment occurs predominantly downstream rather than uniformly surrounding the mutated base. This directional asymmetry distinguishes EMS-induced mutations from spontaneous replication-associated mutations, where G/C enrichment has been reported symmetrically around mutated sites (30, 31), suggesting a fundamentally different mechanism of mutation accrual.

## DISCUSSION

Researchers have made significant advances in the tractable genetic perturbation of obligate intracellular bacteria (4). However, genetic perturbation of diverse intracellular bacterial symbionts remains an extended goal of researchers looking to better understand and harness their unique biological niches. Obligate intracellular symbionts like *Wolbachia* are already being used in large-scale biological control projects seeking to improve both agricultural and human health (1, 2) yet approaches to modify their genomes are lacking. Here, we validated the use and detection of EMS for random chemical mutagenesis of intracellular *w*Mel *Wolbachia* infecting *Drosophila* cell culture. Using an extremely low error rate sequencing method (17), we produced mutation profiles with a nearly fivefold increase above the background mutation and sequencing error rate. Together, these results establish EMS mutagenesis as a tractable and quantifiable approach for generating genome-wide genetic variation in intracellular *Wolbachia*, providing a practical foundation for future functional genetic studies in systems where genetic manipulation has historically been inaccessible.

Beyond establishing the feasibility of detecting EMS-induced mutations in an intracellular genome, our results suggest that EMS imposes a largely uniform mutational burden across functional genomic categories. The minimal difference observed between coding and non-coding mutation rates indicates that EMS does not preferentially target or spare protein-coding regions, consistent with broadly stochastic mutation rather than context-specific targeting or repair. This uniformity is further supported by the absence of extended mutational hot or cold spots across the genome, arguing against large-scale regional variation in EMS accessibility. This implies that, with sufficient mutational saturation, EMS mutagenesis is expected to impact essentially all genomic regions in *Wolbachia*, making it well suited for genome-wide functional interrogation.

At a finer scale, the lack of a strong relationship between gene expression and mutation rate suggests that transcription-coupled processes play a limited role in shaping EMS mutational outcomes across the *w*Mel genome. However, clear sequence-context preferences are evident, with a 5-mer model providing the most informative description of EMS mutation bias. The fact that even this model performs modestly underscores that EMS mutagenesis is influenced by factors beyond local sequence alone, likely reflecting interactions with genome organization or DNA repair pathways. Together, these findings highlight both the predictability and the limits of sequence-based models of EMS mutagenesis in intracellular genomes.

Our work proposes a future for genomic screens in *Wolbachia* as well as the potential within other host-cell cultured endosymbionts. This demonstration of high-confidence detection of EMS-induced mutations in *Wolbachia* using ultra-low error rate sequencing builds on previous models of EMS mutation rate and bias and lays the groundwork for larger screens. A key consideration for future mutagenesis screens will be understanding how EMS-induced mutations are maintained or lost over extended culture periods, a necessary step in translating chemical mutagenesis from a tool for generating variation into one capable of supporting phenotypic screens. Doing so will require the development of dose and recovery experimental frameworks that track mutant frequency over time, building on the quantitative mutational foundation established. Future directions into this phenotypic space will also likely seek to explore correlations between mutation effect, titer, and pathogenicity. Another important consideration for future work is the direct comparison of mutation rates between *in vitro* and *in vivo* conditions, such as Wolbachia infecting D. melanogaster. With EMS mutagenesis and high-confidence mutation detection now established in cell culture, extending this approach to *in vivo* systems becomes a more tractable goal.

## Data Availability

All code used for processing circle sequencing data can be found here: https://github.com/jmcbroome/circleseq. All code used for rate modeling, analysis, and data visualization can be found here: https://github.com/gabepen/ems_effect/tree/manuscript-scope. All raw sequencing data files can be found under NCBI Bioproject PRJNA1427293.

## SUPPLEMENTAL MATERIAL

Supplemental material msystems.00660-26-s0001.docx Supplemental table legends and figures.

Table S1 msystems.00660-26-s0002.xlsx Read counts.

Table S2 msystems.00660-26-s0003.xlsx Per-sample metrics.

Table S3 msystems.00660-26-s0004.xlsx Per-gene metrics.

Table S4 msystems.00660-26-s0005.xlsx Sequence bias model selection values.

Table S5 msystems.00660-26-s0006.xlsx Pentamer mutation rates.

ASM does not own the copyrights to Supplemental Material that may be linked to, or accessed through, an article. The authors have granted ASM a non-exclusive, world-wide license to publish the Supplemental Material files. Please contact the corresponding author directly for reuse.
