# Supplementary material for: EMS mutation and SNP detection in intracellular Wolbachia genomes
Source: mSystems. 2026 Jul 6;11(8):e00660-26. doi: 10.1128/msystems.00660-26 (PMC13483387; doi:10.1128/msystems.00660-26)
Supplement: Supplementary file 1 [file msystems.00660-26-s0001.docx]

**Supplemental Materials**

**Table S1. Read Counts**. Per-sample initial reads, RCA products and total read copies.

**Table S2. Per-sample Metrics**

**Table S3. Per-gene Metrics**

**Table S4. Model Selection Summary Statistics**

**Table S5. 5-mer Sequence Bias Model Rates**

**Figure S1. Single Base Mutation Spectra.** Rates for each single base mutation type indicate no presence of a strand bias in the mutation data, as expected.

**Figure S2. EMS mutation density across the genome**. The bars represent 10,000 bp windows. The gap in mutation density coincides with no sequencing coverage. This is due to the drop out of the octomom gene cluster from the JW18 hosted wMel cell line.

**Figure S3. Circle sequencing error rate decrease.** The error rate of circle sequencing decreases with the incorporation of tandemly duplicated subreads.

**Figure S4. Analysis of EMS Mutation Rates Across the wMel Genome and Genes. A)** Plot of 2500 bp windows that have a significantly higher (red) or lower (blue) mutation rate compared to the overall mean. **B, C)** Linear regression analysis of control and treated rates for genes against their expression levels.

**Figure S5. Sequence Bias Model Evaluation. A-D)** Within sample prediction accuracy metrics for each model type. **E-H)** Out of sample cross-validation metrics for each model type
